# Supplementary material for: Content-rich biological network constructed by mining PubMed abstracts
Source: BMC Bioinformatics. 2004 Oct 8;5:147. doi: 10.1186/1471-2105-5-147 (PMC528731; doi:10.1186/1471-2105-5-147)
Supplement: Additional File 2 — The original results of the above study (non-essential files are deleted to keep the file size under the limit set by BMC bioinformatics). [file 1471-2105-5-147-S2.bz2 › chilibotAdditionalFile2/dip05/479ID8702798E10568/html/IL8RB_CXCL2.html]

 


 **IL8RB** and **CXCL2** 
  
Found 50 abstracts in PubMed, retrieved 05.  
 

 What does Google say? 
 PDF only 
| .edu only 

---

**Interactive relationship** (e.g. stimulation, inhibition, etc)

**Neutral relationship**- The CXCR2  [ **IL8RB** ]  is a GPCR that is activated, amongst the others, by the chemokines CXCL8 IL 8 and  **CXCL2**  GROb  [ **CXCL2** ]  to induce cell chemotaxis.  Ref: 12888558 J Biol ChemJ Biol Chem,

**Non-interactive relationship** (e.g. studied together, co-existance, homology, etc.)

- In summary, we have shown that TII alveolar epithelial cells produce three of the major proinflammatory CXC chemokines, GRO, CINC 2alpha and MIP 2  [ **CXCL2** ] , and their cognate receptor CXCR2  [ **IL8RB** ] .  Ref: 12829448 Am J Respir Cell Mol Biol< MedlineTA>Am J Respir Cell Mol Biol,
- Further, inhibition of the MIP 2  [ **CXCL2** ]  receptor, CXCR2  [ **IL8RB** ] , decreased baseline hepatocyte proliferation and stat 3 expression in the setting of partial hepatectomy.  Ref: 12875976 Am J Pathol, 2003
- Taken together, these data demonstrate that Hem induced priming of PMN not only mediates ALI in the mouse, but also that this process is differentially effected by MIP2  [ **CXCL2** ]  and KC, despite the fact that both signal through CXCR2  [ **IL8RB** ] .  Ref: 12688548 Shock, 2003
